# A protocol for prospective observational study to determine if non-anaemic iron deficiency worsens postoperative outcome in adult patients undergoing elective cardiac surgery: the IDOCS study

**DOI:** 10.1186/s13741-022-00239-2

**Published:** 2022-02-08

**Authors:** Lachlan F. Miles, Vanessa Pac Soo, Sabine Braat, Stephane Heritier, Kate L. Burbury, David A. Story

**Affiliations:** 1 Department of Critical Care, Melbourne Medical School, The University of Melbourne, Melbourne, Australia; 2 Methods and Implementation Support for Clinical and Health research Hub, Melbourne School of Population and Global Health, The University of Melbourne, Melbourne, Australia; 3 Department of Epidemiology and Preventative Medicine, Faculty of Medicine, Nursing and Health Sciences, Monash University, Melbourne, Australia; 4 Sir Peter MacCallum Department of Oncology, Faculty of Medicine, Dentistry and Health Sciences, The University of Melbourne, Melbourne, Australia

**Keywords:** Anaemia, Iron deficiency, Cardiac surgical procedures, Postoperative complications, Patient readmission, Ferritins, Iron, Quality of life

## Abstract

**Background:**

Pre-operative anaemia has been associated independently with worse outcomes after cardiac surgery in adults and is often caused by absolute or functional iron deficiency. Iron deficiency is a continuum ending with anaemia, and therefore it is plausible that pre-operative early or ‘non-anaemic’ iron deficiency may also be associated with worse outcomes in patients undergoing cardiac surgery.

**Methods:**

We have designed a prospective, observational study to determine if there is an association between non-anaemic iron deficiency and worse outcomes after cardiac surgery in adults. Patients without anaemia undergoing elective cardiac surgery will be allocated to an iron-deficient and an iron-replete group based on standard pre-operative blood tests (ferritin, transferrin saturation and C-reactive protein). The primary outcome is days alive and at home on postoperative day 30. The key secondary outcomes are days alive and at home on postoperative day 90 and readmission to acute care. Other secondary outcomes include health-related quality of life questionnaires, quality of postoperative recovery, postoperative complications, changes in haemoglobin concentration, and requirement for allogeneic blood products. The planned study sample size is 240 patients per group, which has 83% power to detect a median difference of 1.25 days in the primary outcome. The study commenced in March 2018, and recently completed recruitment, with data audit and cleaning ongoing.

**Discussion:**

This study will be conducted using a rigorous, prospective observational design; it will provide peak bodies and clinicians with high-quality evidence concerning the associations between non-anaemic iron deficiency and patient-centred outcomes after elective cardiac surgery. Our primary and key secondary outcomes are known to have great importance to clinicians and patients alike and align with the recommendations of the StEP-COMPAC group for outcomes in prospective peri-operative research. The definition used for iron deficiency accounts for both absolute and functional iron deficiency and make use of standard pre-operative blood tests to make this determination, easing the transition of results into clinical practice. The study will be conducted in two relatively high-volume centres in a single high-income country. This limits the generalisability of study results to similar centres.

**Trial registration:**

Australian and New Zealand Clinical Trials Registry (ACTRN12618000185268). Registered 5 February 2018.

**Supplementary Information:**

The online version contains supplementary material available at 10.1186/s13741-022-00239-2.

## Background

Anaemia has been independently associated with worse outcomes after cardiac surgery (Rössler et al., 2020; Klein et al., 2016; Hogan et al., 2015; Miles et al., 2018a). Outside the surgical context, anaemia has been associated with reduced exercise capacity, higher incidence of infection and reduced quality of life (Lopez et al., 2016). In the adult patient undergoing cardiac surgery, pre-operative anaemia is independently associated with increased mortality, increased hospital stay, and increased allogeneic transfusion requirement (Klein et al., 2016). Whilst there are multiple possible causes of reduced pre-operative haemoglobin concentration in this cohort, iron deficiency is most frequently implicated (Hung et al., 2015; Muñoz et al., 2017a; Karski et al., 1999; David et al., 2013). The relationship between iron deficiency and anaemia represents a continuum, with iron deficiency anaemia representing advanced iron deficiency (Miles & Story, 2019). In a recently reported observational study using data previously collected for another prospective randomised trial (Spahn et al., 2019), Rössler et al. articulated this graduated association. In this study, patients who were not iron deficient or anaemic had a mortality of 2%, increasing to 4% in patients with anaemia unrelated to iron deficiency, 5% in patients with non-anaemic iron deficiency (NAID) and 15% in patients with iron deficiency anaemia. The physiology of iron handling provides biological plausibility for this observation—whilst 60% of iron stores are involved in erythropoiesis, a further 30% is integral to respiratory chain function, and 10% is incorporated into myoglobin (Suominen & Punnonen, 1998). By the time iron deficiency is sufficiently advanced to cause anaemia, non-erythroid compartments have already been depleted. It is therefore possible that iron deficiency, in the absence of anaemia, might be associated with worse postoperative outcomes, although not to the same degree as iron deficiency anaemia.

Improved understanding of the role of iron in the inflammatory responses has in turn influenced how iron deficiency is defined (Camaschella & Girelli, 2020). Whilst Rössler et al. defined iron deficiency using serum ferritin in isolation (ferritin < 100 μg/L), this approach will result theoretically in some patients with iron deficiency being mischaracterised as iron replete. Several trials in the heart failure literature have defined iron deficiency using multiple different categorisations, taking into account the phenomenon of functional iron deficiency, and recognising the role of ferritin as an acute phase reactant and possible active participant in inflammation (Anker et al., 2009; Van Veldhuisen et al., 2017; Okonko et al., 2008; Kell & Pretorius, 2014; Fitzsimons & Doughty, 2015).

Broadly speaking, iron deficiency is presently defined as:

- Absolute iron deficiency—a true deficiency in stored iron, commonly due to inadequate intake or excessive loss. Commonly defined as a serum ferritin < 30 μg/L.
- Functional iron deficiency—inability for apparently adequate iron stores to cross basement membranes due to increased expression of the iron regulatory hormone hepcidin in response to inflammation. Commonly defined as a serum ferritin between 100 and 300 μg/L with a transferrin saturation (TSAT) < 20%, indicating increased bodily iron demand.
- Inadequate iron stores—describes a situation where serum ferritin would normally be adequate, but may become inadequate were the patient to face a major inflammatory stressor or blood loss (Muñoz et al., 2017b). Commonly defined as serum ferritin < 100 μg/L in the context of upcoming major surgery.

Prior to the publication of Rössler et al., consensus statements and best practice guidelines recommended routine correction of NAID prior to major surgery using a variety of different criteria including, in some cases, variant definitions of functional iron deficiency (Muñoz et al., 2017b; National Blood Authority, 2012). This is despite the absence of direct prospective evidence that functional iron deficiency without anaemia is associated with worse outcomes after major surgery, and despite the absence of robust randomised controlled trial data to support routine correction of NAID prior to major surgery (Miles et al., 2019). These distinctions are important—despite the safety of newer iron preparations (Avni et al., 2015), iron replacement has been hypothesised to increase the risk of infection (Litton et al., 2013). Whilst retrospective and registry studies are compelling (Muñoz et al., 2014), a clear association between NAID, particularly with respect to functional iron deficiency, and worse outcomes after cardiac surgery should be established, and provide equipoise for subsequent clinical trials aiming to assess the effect of pre-operative iron therapy in this population.

We hypothesise that adult patients undergoing elective cardiac surgery with iron deficiency but not anaemia will have a worse postoperative outcome relative to patients who are iron replete without anaemia. We therefore aim to assess if NAID is associated with a reduction in days alive and at home on postoperative day 30 (DAH_30_) in adult patients undergoing elective cardiac surgery, relative to those patients who are neither anaemic nor iron deficient. DAH_30_ is a composite, patient-centred outcome measure of inpatient stay, readmission, rehabilitation requirements, complications and mortality (Myles et al., 2017). Additionally, secondary outcomes will be used to determine whether NAID actively worsens outcome or recovery after surgery, as measured by a variety of laboratory and additional patient-centred quality of life metrics, as well as the requirement for allogeneic blood transfusion. Accordingly, this study will use a cohort of 480 patients, and a prospective, observational, multi-centre design to assess these associations. Participants will be divided into iron-deficient (*n* ≈ 240) and iron-replete (*n* ≈ 240) groups, based on the results of standard preoperative testing.

## Methods/design

The study will be performed at two tertiary, university-affiliated centres in Melbourne, Australia, with an annual cardiac surgery case load of at least 350 cases.

### Inclusion/exclusion criteria

Adult patients undergoing elective cardiac surgery will be eligible for screening. Patients requiring urgent or emergency surgery as per the EuroSCORE-II criteria are excluded from the study (Nashef et al., 2012). Many patients requiring urgent or emergency cardiac surgery will have required coronary angiography in the week prior to operation. Angiography has been associated with haemoglobin loss and increased transfusion requirement in patients who have subsequently required urgent or emergency surgery. Therefore, there is a risk that patients requiring such surgery are more likely to be classified as iron deficient, introducing selection bias (Ereth et al., 2000). Patients will also be excluded if they are pregnant, below the age of 18 years, have received iron or erythropoiesis-stimulating agents (ESAs) in the month prior to surgery, have end-stage renal failure requiring dialysis (because of the high incidence of iron deficiency in this population), have a known or suspected haemoglobinopathy, bone marrow disease or haemochromatosis. Patients with anaemia as defined by the current World Health Organization criteria will also be excluded (World Health Organization, 2011). Co-recruitment into other observational or interventional studies is permitted unless the intervention involves exogenous iron or ESAs. Participants should not receive exogenous iron or ESAs until 90-day follow-up is completed. If a patient receives exogenous iron or ESAs, their data will still be included in the analysis. The number of patients in each group who received intravenous iron against study protocol will be reported.

The ratio of iron-deficient to iron-replete patients will be monitored throughout the study. Once 240 participants from one group have been entered into the database, recruitment to this group will cease. Recruitment to the other group will continue until the planned enrolment of 480 patients is reached.

### Assessments

Baseline laboratory assessment will be performed using standard pre-operative blood tests (full blood examination [FBE], serum ferritin, TSAT, urea and electrolytes, liver function tests, coagulation profile and C-reactive protein [CRP]) as recommended by the Australian National Blood Authority (National Blood Authority, 2012). Glomerular filtration rate (GFR) will be estimated using the Chronic Kidney Disease Epidemiology equation (Levey et al., 2009). Results will be acceptable for stratification and screening if they are taken within 90 days of the index surgical procedure. A central laboratory will not be used, and the instrument upon which study blood tests are performed will not be controlled for beyond standard calibration as required by the National Association of Testing Authorities, Australia. Additional baseline demographic data collected will include concomitant medications, smoking, dietary, ethnicity and alcohol status. Clinical data will be collected on the day of surgery prior to induction of anaesthesia, including height, weight, vital signs, and heart rhythm. The schedule of assessments is in Table 1. Patients will be allocated to iron-deficient or iron-replete groups according to preoperative iron studies. A broad definition will be used, taken from studies in the heart failure literature (Fitzsimons & Doughty, 2015).

**Table 1:** Schedule of assessments during the study

Assessment	Pre-anaesthetic assessment	Day of surgery	Intra-operative	Day 3 post-operative	Discharge from acute care	Day 30 postoperative	Day 45 postoperative*	Day 90 postoperative
Eligibility criteria	×							
Patient characteristics		×						
Medical history		×						
EQ-5D-5L		×				×		
WHODAS 2.0		×						×
FBE	×			×			×	
Iron studies	×							
CRP	×							
EuroSCORE-II		×						
Intraoperative data			×					
Requirement for allogeneic blood products			×	×	×	×		
QoR-15				×				
Primary endpoint						×		
Secondary endpoint of special significance								×
Mortality/readmission					×	×		×
Complications					×	×		×
Patient off study								×

*WHODAS* World Health Organization Disability Assessment Schedule, *FBE* full blood examination, *CRP* C-reactive protein, *EuroSCORE* European System for Cardiac Operative Risk Evaluation, *QoR* quality of recovery

*Assessment performed in one centre only

Iron deficiency will be defined as:

- Ferritin < 100 μg/L;
- Ferritin 100–300 μg/L and CRP > 5 mg/L; or
- Ferritin 100–300 μg/L and TSAT < 20%.

Iron repletion will be defined as:

- Ferritin > 300 μg/L; or
- Ferritin 100–300 μg/L and CRP ≤ 5 mg/L and TSAT > 20%.

### Outcomes

The primary outcome will be DAH_30_. A secondary outcome measure of special importance will be DAH_90_. Additional secondary outcomes measures will be duration of acute hospital stay, duration of intensive care admission during initial hospital stay, requirement for readmission to acute care within the first 90 days postoperatively, requirement for allogeneic blood transfusion, and haemoglobin concentration relative to baseline measured on postoperative day 3, acute care discharge (or closest approximate value), and at one of the two participating centres on postoperative day 45.

Health-related quality of life outcomes to be assessed are functional status at baseline and postoperative day 30 as measured by the 5-level European Quality of Life Score (EQ-5D-5L) questionnaire (Herdman et al., 2011), disability status at baseline and postoperative day 90 as measured by the World Health Organization Disability Assessment Schedule (WHODAS 2.0) questionnaire (Shulman et al., 2015), and recovery from surgery on postoperative day 3 as measured by the 15-item Quality of Recovery (QoR-15) questionnaire (Stark et al., 2013).

As the study does not involve an intervention, safety and efficacy related outcomes will not be measured per se. However, postoperative complications (including death) have been defined a priori by system using clinical and laboratory criteria and will be reported as part of the study, effectively serving as a safety endpoint (Appendix 1).

All study results will be reported in keeping with the Strengthening the Reporting of Observational Studies in Epidemiology (STROBE) guidelines (von Elm et al., 2007).

### Sample size and justification

Data assumptions for the primary outcome were obtained from a retrospective review of similar cases at one of the participating hospitals (Miles et al., 2018b). Whilst DAH_30_ was not explicitly reported as part of this dataset, it was obtained on re-analysis. The primary endpoint (DAH) has a left-skewed distribution with a spike at 0, mainly due to deaths in hospital post-surgery. Power was obtained by simulations (20,000). DAH-looking data were generated by simulating first length of stay using a lognormal distribution, truncating it at 28 days (a plausible maximum based on existing data) and finally computing DAH as length of stay (days) subtracted from 28. Such data are bimodal with a spike at 0 as required. The lognormal distribution was calibrated to generate DAH with a median of 23 days and 5% zeroes in the NAID group. A sample size of *n* = 480 (240/group) participants has 83% power to detect a median increase of 1.25 days using the Wilcoxon sum rank test for the analysis and allowing for up to 5% dropout in each group. Sample size calculations were performed in R 3.4.0 (R Foundation for Statistical Computing, Vienna, Austria).

### Statistical analysis plan

The analysis will include all participants enrolled in the study. Summary statistics will consist of counts and percentages for categorical variables, mean and standard deviation for continuous variables, or median and quartiles (25th and 75th percentiles) for non-symmetrical continuous variables.

The primary outcome, DAH_30_, will be modelled using median or, more generally, quantile regression (Koenker & Bassett, 1978). As DAH_30_ is left skewed with a spike at zero, it is more relevant to model the median that is closer to the major distribution mode and directly interpretable (Koenker & Bassett, 1978). Difference in median DAH_30_ between the two groups (patients with iron deficiency versus those who are iron replete) will be obtained unadjusted (including recruiting hospital) and adjusted for potential known confounders, namely EuroSCORE-II and body surface area (in addition to recruiting hospital). A similar strategy will be used for DAH_90_. In addition, a propensity score analysis will be carried out. Using logistic regression, the probability (propensity score) that a participant would have been allocated to the iron-deficient or iron-replete group will be estimated by including in the model potential confounding baseline characteristics (EuroSCORE-II, diet and CRP). This propensity score will be used as a covariate in adjusting the quantile regression model with the group variable and hospital. Balance and underlying assumptions (i.e. linearity and no interaction between the group and propensity score) will be checked.

Additional secondary binary outcomes will be analysed using logistic regression and continuous outcomes using linear regression adjusted for the baseline reading where applicable. A repeated measures analysis based on mixed linear models will also be conducted for blood haemoglobin. Time-to-event will be analysed using Cox proportional hazards regression. Safety endpoints and postoperative complications that exhibit small numbers will be analysed using exact logistic regression. All statistical analyses will be conducted both unadjusted (including hospital) and adjusted for potential known confounders (in addition to hospital).

Subgroup analyses will be performed to explore heterogeneity in the effect between centres, sex, age groups and estimated glomerular filtration rate grouping by including a term for subgroup and interaction between subgroup and group in the models. An exploratory analysis will be performed stratifying participants by sex and haemoglobin strata (130–139 g/L and ≥ 140 g/L for men, and 120–129 g/L, 130–139 g/L and ≥ 140 g/L for women). Finally, additional analyses will be performed based on different definitions of iron deficiency used in the literature previously (TSAT < 20% vs. TSAT ≥ 20% and serum ferritin < 100 μg/L vs. ≥ 100 μg/L) (Richards et al., 2020). The outcomes analysed will be DAH_30_, DAH_90_, readmission up to postoperative day 90 and postoperative complications (including infection). Analyses will use complete cases. If the proportion of missing data is above 5% (Jakobsen et al., 2017), additional analysis using inverse probability weighting will be used where complete cases will be weighted by the inverse of their probability of being a complete case (SR S, IR W., 2013).

All statistical tests will be performed, and confidence intervals reported at the two-sided 5% level of significance. To control the overall Type I error rate across DAH_30_ and DAH_90_, a hierarchical testing strategy is planned where the null hypothesis of no difference in median DAH_90_ will be tested at the two-sided 5% significance level only if the null hypothesis of no difference in median DAH_30_ is rejected at the two-sided 5% significance level. All analyses will be carried out in Stata 16.1 (Stata Corporation, College Station, TX, USA).

### Data management, storage, and security

Data will be entered initially into a hard copy case report form and transcribed subsequently into a secure, electronic database (REDCap v11.1.8, Vanderbilt University, TN, USA). Access to both hard copy and electronic data will be limited to study personnel. Study data will be de-identified, with a master linking log enabling re-identification stored separately and accessible only to site investigators.

### Patient and public involvement

There was no patient or public involvement in the design of the research. However, patient-centred outcome measures are integral to the study design.

## Ethics and dissemination

The trial was prospectively registered on the Australian and New Zealand Clinical Trials Registry (ACTRN126180002185268). Ethics approval was granted by the Austin Health Human Research Ethics Committee (HREC/Austin/16/308).

## Current status of the trial

The first patient was recruited on 26 March 2018. Enrolment was completed in the iron-deficient group (*n* = 240) on 6 January 2021, and in the iron-replete group (*n* = 240) on 11 February 2021. Participant follow-up concluded in May 2021. Aggregate characteristic data from the 480 participants enrolled in the study are shown in Table 2.

**Table 2:** Aggregate pre-operative characteristics of the study cohort

Characteristics	Value (***n*** = 480)
Female	95 (19.7%)
Age (years)	64.1 (11.6)
Height (m)	1.72 (0.09)
Weight (kg)	88.4 (17.0)
BMI (kg/m^2^)	29.7 (5.1)
BSA (m^2^)	2.04 (0.23)
Previous MI	72 (15%)
Congestive cardiac failure	112 (23.3%)
Peripheral vascular disease	24 (5%)
COPD	49 (10.2%)
Diabetes mellitus	118 (24.5%)
Chronic renal impairment	44 (9.2%)
Active smoker	75 (15.6%)
EuroSCORE-II (%)	1.08 (0.69–1.74)
LVEF > 50%	417 (86.9%)
LVEF 41–50%	54 (11.3%)
LVEF 31–40%	6 (1.2%)
LVEF ≤ 30%	3 (0.6%)
Pre-operative PASP ≥ 31 mmHg	45 (9.3%)

*BMI* body mass index, *BSA* body surface area, *MI* myocardial infarction, *COPD* chronic obstructive pulmonary disease, *EuroSCORE* European System for Cardiac Operative Risk Evaluation, *LVEF* left ventricular ejection fraction, *PASP* pulmonary artery systolic pressure

The postoperative day 45 haemoglobin will not be reported in the primary manuscript at this stage, and instead will be the subject of an additional hypothesis-generating analysis examining specific secondary outcomes (DAH_90_ and readmission). At the time of writing, formal database lock and analysis has not yet been performed.

## Discussion

Increasing attention is being paid to the continuum of iron deficiency and anaemia in patients undergoing cardiac surgery(Rössler et al., 2020; Miles et al., 2018a; Immohr et al., 2020; Piednoir et al., 2011; Hubert et al., 2019). However, despite recommendations from peak bodies (National Blood Authority, 2012), iron status is only viewed as a therapeutic target by many clinicians once anaemia has supervened. This is despite the observation that end-organ manifestations of iron deficiency may be present prior to anaemia becoming apparent (Favrat et al., 2014). However, whilst there is biological plausibility that NAID may be associated with worse postoperative outcomes, the existing evidence is either retrospective in nature (Miles et al., 2018b), or uses a definition of iron deficiency inconsistent with most modern RCTs (Rössler et al., 2020; Piednoir et al., 2011). Despite the absence of a firmly established association between NAID and outcome after cardiac surgery, there are consensus statements that recommend both functional and absolute iron deficiency be corrected pre-operatively (Muñoz et al., 2017b), supported, in part, by underpowered subgroup analyses of small randomised trials (Johansson et al., 2015), or randomised trials that incorporate therapies other than iron (Spahn et al., 2019). Critically, the evidence for this practice suffers from indirectness and was graded as low or very low in a recent systematic review (Miles et al., 2019). Whilst newer iron preparations have a vastly improved safety profile relative to historic versions of the drug (Avni et al., 2015), there are still theoretical concerns that iron therapy may increase the risk of infection (Litton et al., 2013), and specific preparations such as ferric carboxymaltose have been associated with biochemical hypophosphataemia (Fang et al., 2019; Wolf et al., 2020). Additionally, unnecessary iron treatment may delay surgery, and has health economic implications. The IDOCS study was designed with these conundrums in mind and aims to determine if there is a firm, prospectively assessed association between NAID and negative outcome after cardiac surgery. If such a relationship cannot be established, then it is difficult to justify emerging recommendations that NAID should be routinely treated prior to cardiac surgery.

The study design is pragmatic, and wherever possible uses standard of care tests as recommended by the National Blood Authority to improve the generalisability of any results (National Blood Authority, 2012). However, we acknowledge that as this study is conducted in two centres in a single high-income country, the global generalisability of results will be limited. Primary and key secondary outcomes are known to have great importance to clinicians and patients alike and align with the recommendations of the StEP-COMPAC group for outcomes in prospective peri-operative research (Haller et al., 2019). Whilst this study is conceptually related to patient blood management (Pagano et al., 2018), it should be noted that the primary objective of the study is not to examine associations between NAID and unnecessary blood transfusion, but to examine the effect of NAID on patient-centred outcome metrics. Patients with NAID are less likely to require transfusion relative to patients with iron deficiency anaemia (Miles et al., 2020), largely due to the higher starting haemoglobin concentration and the associations between anaemia and comorbid conditions (Saager et al., 2013; Klein et al., 2017). Nevertheless, transfusion requirement remains an important secondary outcome measured in this study.

## Supplementary Information

**Additional file 1.** Appendix 1. Prespecified definitions for postoperative complications (IDOCS_Appendix 1.docx).

## Data Availability

All data relevant to the study protocol is included as part of this manuscript or as an Appendix. The prospective listing of the study on the Australian and New Zealand Clinical Trials Registry can be found at https://www.anzctr.org.au/Trial/Registration/TrialReview.aspx?id=374387.

## Abbreviations

CRP: C-reactive protein
DAH: Days alive and at home
DAH_30_: Days alive and at home on postoperative day 30
DAH_90_: Days alive and at home on postoperative day 90
EQ-5D-5L: Five-level European Quality of life score
ESA: Erythropoiesis-stimulating agent
FBE: Full blood examination
GFR: Glomerular filtration rate
NAID: Non-anaemic iron deficiency
QoR-15: 15-item quality of recovery score
StEP-COMPAC: Standardised endpoints in perioperative medicine – core outcome measures in perioperative and anaesthetic care
STROBE: Strengthening the reporting of observational studies in epidemiology
TSAT: Transferrin saturation
WHODAS 2.0: World Health Organization disability assessment schedule 2.0
