# Supplementary material for: A protocol for prospective observational study to determine if non-anaemic iron deficiency worsens postoperative outcome in adult patients undergoing elective cardiac surgery: the IDOCS study
Source: Perioper Med (Lond). 2022 Feb 8;11:4. doi: 10.1186/s13741-022-00239-2 (PMC8822844; doi:10.1186/s13741-022-00239-2)
Supplement: Supplementary file 1 — Additional file 1. Appendix 1. Prespecified definitions for postoperative complications (IDOCS_Appendix 1.docx). [file 13741_2022_239_MOESM1_ESM.docx]

**Appendix 1 – Complication definitions**

1. **Death**

All-cause mortality at hospital discharge, 30 days, and up to 90 days post-surgery.

Evidence of the cause of death to be kept on file at the participating site.

1. **Infection**

Defined by the Centers for Disease Control and Prevention (CDC) with the National Healthcare safety Network (NHSN) criteria.

| Surgical Site Infection (SSI) | |
| --- | --- |
| Superficial SSI | Infection occurs within 30 days after the operative procedure **AND**  involves only skin and subcutaneous tissue of the incision **AND**  patient has at least **one** of the following:  a. purulent drainage from the superficial incision  b. organisms identified from a specimen from the superficial incision or subcutaneous tissue by a culture  c. superficial incision that is deliberately opened by a medical practitioner **AND**  patient has at least one of the following signs or symptoms:  pain or tenderness; localized swelling; erythema; or heat  d. diagnosis of a superficial incisional SSI by a medical practitioner. |
| Deep incisional SSI | Infection occurs within 30 or 90 days after the operative procedure **AND**  involves deep soft tissues of the incision **AND**  patient has at least one of the following:   1. purulent drainage from the deep incision 2. a deep incision that spontaneously dehisces, or is deliberately opened or aspirated by a medical practitioner and organism is identified by a culture **AND**   patient has at least ***one*** of the following signs or symptoms: fever (>38°C); localized pain or tenderness  c. an abscess or other evidence of infection involving the deep incision that is detected on gross anatomical or histopathologic exam, or imaging test. |
| Organ space | Infection occurs within 30 or 90 days after the operative procedure **AND**  involves any part of the body deeper than the fascial/muscle layers, that is opened or manipulated during the operative procedure **AND**  patient has at least ***one*** of the following:  a. purulent drainage from a drain that is placed into the organ/space  b. organisms are identified from an aseptically obtained fluid or tissue in the organ/space by a culture  c. an abscess or other evidence of infection involving the organ/space that is detected on gross anatomical or histopathologic exam, or imaging test evidence suggestive of infection**.** |

| Blood stream |
| --- |
| Patient has an organism identified from a blood culture and that organism is not related to an infection at another site. |

| UTI |
| --- |
| 1. Patient has at least one of the following signs or symptoms:  - fever (> 38.0°C) - suprapubic tenderness - costovertebral angle pain or tenderness - urinary urgency - urinary frequency - dysuria   AND   1. Patient has a urine culture with no more than two species of organisms identified, at least one of which is a bacterium of ≥ 10^5^ CFU.ml^-1^ |

| Pneumonia |
| --- |
| Defined by imaging or signs/symptoms or laboratory tests as documented below:   1. Two or more serial chest imaging results with at least one of the following:  - New and persistent or progressive and persistent   - infiltrate or   - consolidation or   - cavitation   *Note: In patients without underlying pulmonary or cardiac disease, one definitive imaging test result is acceptable.*   1. For any patient, at least one of the following:  - Fever > 38.0°C - Leukopenia (≤ 4000 WBC.mm^-3^) or leukocytosis (> 12,000 WBC.mm^-3^) - For adults > 70 years old, altered mental status with no other recognized cause   AND at least two of the following:   - - New onset of purulent sputum or change in character of sputum, or increased respiratory secretions, or increased suctioning requirements   - New onset or worsening cough, or dyspnea, or tachypnea   - Crackles or bronchial breath sounds   - Worsening gas exchange (e.g. O_2_ desaturations [e.g. PaO_2_/FiO_2_ ≤ 240], increased oxygen requirements, or increased ventilator demand)  1. Positive culture from respiratory secretions or tissue. |

| Sepsis |
| --- |
| Confirmed or presumed infection plus Systemic Inflammatory Response Syndrome (SIRS) ≥2 criteria:   1. T > 38ºC or < 36ºC 2. WCC > 12 or > 10% immature band forms 3. HR > 90/min 4. RR > 20/min or PaCO_2_ < 32mmHg |

1. **Cardiovascular**

| Myocardial infarction - defined by the European Society of Cardiology, American College of Cardiology Foundation, American Heart Association and the World Heart Federation Taskforce universal definition | |
| --- | --- |
| Acute MI | Any one of the following criteria meets the diagnosis for acute MI:   1. Detection of a rise and/or fall of troponin with at least one value above the 99th percentile upper reference limit and with at least one of the following:    - Symptoms of ischaemia.    - New or presumed new significant ST-segment–T wave (ST–T) changes or new left bundle branch block (LBBB).    - Development of pathological Q waves in the ECG.    - Imaging evidence of new loss of viable myocardium or new regional wall motion abnormality.    - Identification of an intracoronary thrombus by angiography or autopsy. 2. Cardiac death with symptoms suggestive of myocardial ischaemia and presumed new ischaemic ECG changes or new LBBB, but death occurred before cardiac biomarkers were obtained, or before cardiac biomarker values would be increased. |
| Prior MI | Any one of the following criteria meets the diagnosis for prior MI:   1. Pathological Q waves with or without symptoms in the absence of non-ischaemic causes. 2. Imaging evidence of a region of loss of viable myocardium that is thinned and fails to contract, in the absence of a non-ischaemic cause. 3. Pathological findings of a prior MI. |

| Heart failure/pulmonary oedema |
| --- |
| Symptoms of heart failure, typically breathlessness or fatigue, either at rest or during exertion, or ankle swelling and objective evidence of cardiac dysfunction at rest.[^67^](#_ENREF_67) |

| Thromboembolism | |
| --- | --- |
| DVT | Evidence of occlusion of a peripheral artery consistent with either an acute local thrombotic event or a peripheral arterial embolism. Findings by either surgical report, pathological specimen, imaging or autopsy. |
| PE | - - - - VQ scan showing high probability of emboli, **OR**       - pulmonary angiogram or spiral CT |

| Arrhythmia |
| --- |
| New postoperative arrhythmia (after patient leaves the operating theatre) requiring drug therapy (rate or rhythm control), DC reversion or permanent pacemaker insertion (i.e., atrial fibrillation, atrial flutter, ventricular tachycardia, ventricular fibrillation, complete heart block, etc.). |

1. **Respiratory**

- Respiratory depression requiring drug treatment or other intervention
- Pneumonia (see above)

1. **Neurological**

- Delirium, hallucinations, seizures
- CVA: new cerebral infarction or haemorrhage on CT or MRI scan, or new neurological signs (paralysis, weakness or speech difficulties) lasting more than 24 hours or leading to earlier death.

1. **Gastrointestinal**

- Ileus
- Small bowel obstruction

1. **Urinary System**

Acute kidney injury – RIFLE criteria

| Class | GFR | UO |
| --- | --- | --- |
| Risk | ↑ SCr × 1.5 or ↓ GFR >25% | <0.5 ml.kg^-1^.h^-1^ × 6 h |
| Injury | ↑ SCr × 2 or ↓ GFR >50% | <0.5 ml.kg^-1.^h^-1^ × 12 h |
| Failure | ↑ SCr × 3 or ↓ GFR >75% or if baseline SCr ≥ 353.6 μmol.l^-1^ (≥4 mg.dl^-1^) ↑ SCr > 44.2 μmol.l^-1^ (> 0.5 mg.dl^-1^) | <0.3 ml.kg^-1^.h^-1^ × 24 h or anuria × 12 h |
| Loss of kidney function | Complete loss of kidney function > 4 weeks | |
| End stage kidney disease | Complete loss of kidney function > 3 months | |

*GFR = glomerular filtration rate; UO = urine output; SCr = serum creatinine.*

1. **Skin**

- Rashes

1. **Musculoskeletal**

- Injury/falls

1. **Re-admission**

- Re-admission to an acute care hospital after discharge home or to a sub-acute care facility after index surgery.
